# Role of Adiponectin in Cardiovascular Diseases Related to Glucose and Lipid Metabolism Disorders

**DOI:** 10.3390/ijms232415627

**Published:** 2022-12-09

**Authors:** Wen Han, Shuxian Yang, Haiyan Xiao, Min Wang, Jingxue Ye, Li Cao, Guibo Sun

**Affiliations:** 1 Beijing Key Laboratory of Innovative Drug Discovery of Traditional Chinese Medicine (Natural Medicine) and Translational Medicine, Institute of Medicinal Plant Development, Chinese Academy of Medical Sciences & Peking Union Medical College, Beijing 100193, China; 2 Key Laboratory of Bioactive Substances and Resources Utilization of Chinese Herbal Medicine, Ministry of Education, Institute of Medicinal Plant Development, Chinese Academy of Medical Sciences & Peking Union Medical College, Beijing 100193, China; 3 Key Laboratory of Efficacy Evaluation of Chinese Medicine against Glycolipid Metabolic Disorders, State Administration of Traditional Chinese Medicine, Institute of Medicinal Plant Development, Chinese Academy of Medical Sciences & Peking Union Medical College, Beijing 100193, China; 4 National Medical Products Administration Key Laboratory for Research and Evaluation of Pharmacovigilance, Beijing 100193, China; 5 Key Laboratory of New Drug Discovery Based on Classic Chinese Medicine Prescription, Chinese Academy of Medical Sciences & Peking Union Medical College, Beijing 100193, China

**Keywords:** adiponectin, glucose and lipid metabolism, atherosclerosis, hypertension, cardiac hypertrophy, myocardial infarction, myocardial ischemia/reperfusion injury

## Abstract

Lifestyle changes have led to increased incidence of cardiovascular disease (CVD); therefore, potential targets against CVD should be explored to mitigate its risks. Adiponectin (APN), an adipokine secreted by adipose tissue, has numerous beneficial effects against CVD related to glucose and lipid metabolism disorders, including regulation of glucose and lipid metabolism, increasing insulin sensitivity, reduction of oxidative stress and inflammation, protection of myocardial cells, and improvement in endothelial cell function. These effects demonstrate the anti-atherosclerotic and antihypertensive properties of APN, which could aid in improving myocardial hypertrophy, and reducing myocardial ischemia/reperfusion (MI/R) injury and myocardial infarction. APN can also be used for diagnosing and predicting heart failure. This review summarizes and discusses the role of APN in the treatment of CVD related to glucose and lipid metabolism disorders, and explores future APN research directions and clinical application prospects. Future studies should elucidate the signaling pathway network of APN cardiovascular protective effects, which will facilitate clinical trials targeting APN for CVD treatment in a clinical setting.

## 1. Introduction

Cardiovascular disease (CVD) is the main cause of death and disease burden globally. The proportion of CVD-related deaths has steadily increased from 12.1 million in 1990 to 18.6 million in 2019 [1]. Glucose and lipid metabolism disorders are important causes of CVD. Indeed, studies have demonstrated that insulin resistance (IR), hyperglycemia, and dyslipidemia increase cardiovascular morbidity and mortality [2]. Abnormal glucose and lipid metabolism is caused by a deficiency of insulin and IR [3]. IR is an impaired response to insulin stimulation of target tissues, particularly the liver, muscle, and adipose tissue [4]. The heart and blood vessels are surrounded by adipose tissue; the adipose tissue around the epicardium and blood vessels can secrete active lipids, adipokines, or adipocytokines, directly regulating the cardiovascular system [5]. Certain adipokines, such as adiponectin (APN), improve CVD by regulating glucose and lipid metabolism, thereby reducing inflammation and oxidative stress, and facilitating cardiovascular homeostasis [6]. APN-mediated regulation of glucose and lipid metabolism suggests that APN is an insulin-sensitized adipokine, and the APN and insulin signaling pathways converge on the adaptor protein containing pleckstrin homology domain (APPL1), which enhances insulin action and secretion by fine-tuning protein kinase B (Akt) activity in multiple insulin targeting tissues [5]. APN plays a key role in mitigating CVD, especially in cases associated with abnormal glucose and lipid metabolism, including atherosclerosis [7], hypertension [8], and myocardial hypertrophy [9]. APN treatment improves MI-R injury [10] and myocardial infarction [11], and is a potential diagnostic and prognostic biomarker of heart failure (HF) [12].

Based on the important effect of APN on CVD, related to glucose and lipid metabolism disorders, this article reviews its role in these diseases and related advances in recent years, discusses its development prospects, and provides novel perspectives that could facilitate CVD treatment and related drug research and development.

## 2. APN Structure, Receptors, and Signaling Pathways

### 2.1. APN Structure

**Figure 1:**
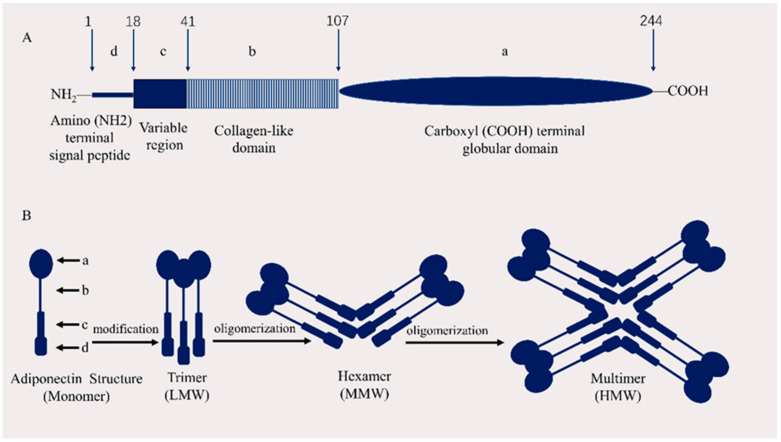
(**A**) Domain structure of human adiponectin (APN). APN monomer is composed of a carboxyl (COOH) terminal globular domain (a), a collagen-like domain (b), a variable region, and (c) an amino (NH2) terminal signal peptide (d) [15] (**B**) APN structure. (**B**) a–d is the same as (**A**) a–d. Three APN monomers are connected to form a trimer (low molecular weight [LMW]), two trimers are connected to form a hexamer (medium molecular weight [MMW]), and 4–6 trimers form multimers (high molecular weight [HMW]) [14,18].

APN, which is composed of 244 amino acids and is encoded by the *ADIPOQ* gene on chromosome 3q27, is a human protein with a molecular weight of 30 kDa and consists of three exons and two introns [13]. APN monomer consists of a carboxyl (COOH) terminal globular domain, a collagen-like domain, a variable region, and an amino (NH2) terminal signal peptide [14] (Figure 1). APN is structurally similar to complement C1q and is also known as Arcp30, AdipoQ, or apM1 [15]. APN is composed of monomers; three APN monomers are linked to form a trimer, and 4–6 trimers are combined to form a high molecular structure. APN monomers are only present in adipocytes and have been reported in the plasma, where they are secreted after forming multimers [16]. Plasma APN contents in healthy individuals range from 2 to 20 mg/L, accounting for approximately 0.01% of the total human plasma protein [15]. It contains a collagen repeat domain at the N-terminus, and a globular domain at the C-terminus. Globular APN (gAcrp), the direct presence of APN in the form of a C-terminal globular domain, is a major region via which APN achieves biological effects [17].

### 2.2. APN Receptors

At present, three APN receptors are known [19]; these include APN Receptor 1 (AdipoR1), APN Receptor 2 (AdipoR2) [20], and T-cadherin [21]. Yamauchi et al. isolated cDNAs encoding the APN receptors AdipoR1 and AdipoR2, demonstrating that they act as globular and full-length APN receptors and mediate increased AMPK and peroxisome proliferator-activated receptor-α (PPAR-α) ligand activity, fatty acid oxidation, and glucose uptake [20]. Hug et al. identified T-cadherin as a hexameric and multimeric APN receptor, but not trimeric or globular. T-cadherin is a glycosylphosphatidylinositol-anchored extracellular protein through which APN transmits metabolic signals [21].

### 2.3. Signaling Pathways of APN

#### 2.3.1. AMPK and PPAR Signaling Pathways

Globular and full-length APN activate AdipoR1 and AdipoR2 [15]. APPL1 binds to AdipoR1 and AdipoR2 intracellular regions through its C-terminal PTB and CC domains [22], mediating AMPK activation downstream effects such as improving glucose uptake by inhibiting Akt/mTOR to affect the insulin signaling pathway [23]. In addition to directly mediating AMPK expression, APPL1 can also directly mediate PPAR-α expression associated with fatty acid oxidation or indirectly activate PPAR-α through AMPK, initiate the downstream β-oxidation pathway, inhibit the activity of acetyl-CoA carboxylase (ACC), increase fatty acid oxidation, regulate lipid metabolism, and reduce oxidative stress [24,25,26,27]. In addition to the effects associated with glucose and lipid metabolism described above, activation of the downstream effects of AMPK are reflected in endothelial cell function enhancement and cardiovascular homeostasis maintenance via increased eNOS activity and nitrous oxide (NO) production via eNOS phosphorylation [28,29]; reduced cardiac hypertrophy via ERK inhibition [30]; activated SIRT1-PGC-1α pathway to promote mitochondrial biogenesis [31,32]; and reduced inflammatory responses via NF-κB/TNF-α pathway inhibition [26].

#### 2.3.2. Akt Signaling Pathway and MAPK Signaling Pathway

**Figure 2:**
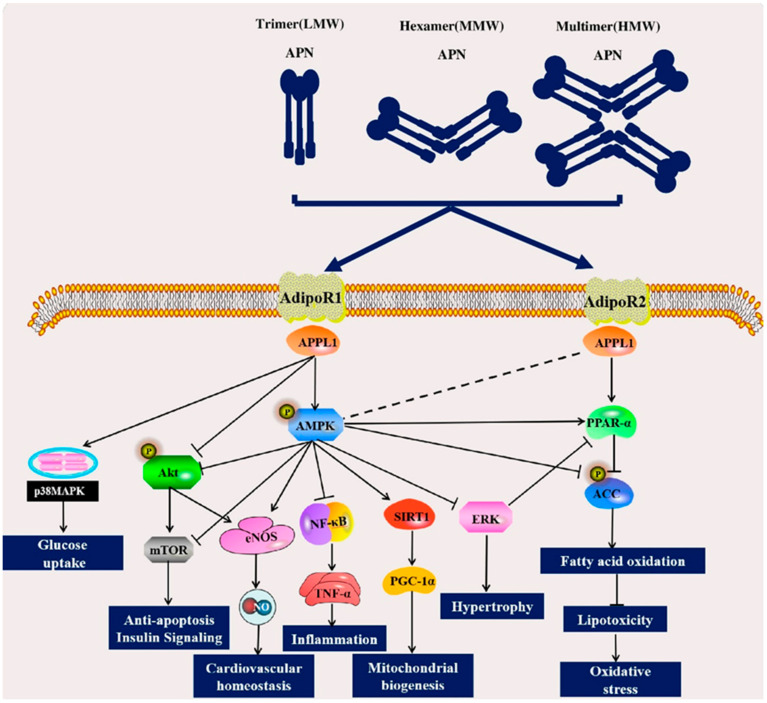
Schematic representation of the intracellular signaling pathways involving adiponectin (APN). APN binds to its receptors AdipoR1 and AdipoR2 and interacts with APPL1, thereby activating various signaling pathways, including AMPK, PPAR-α, and Akt pathways. Activation of these pathways leads to cellular responses, including glucose uptake stimulation, fatty acid oxidation, increased insulin sensitivity, and mitochondrial biogenesis, maintenance of cardiovascular homeostasis, reduction in inflammation, cardiac hypertrophy, and oxidative stress. Black arrows indicate activation, and flat lines indicate inhibition. Abbreviations: ACC, acetyl-CoA carboxylase; AdipoR1, adiponectin receptor 1; AdipoR2, adiponectin receptor 2; AMPK, 5′-adenosine monophosphate-activated protein kinase; Akt, protein kinase B; APPL1, adaptor protein containing pleckstrin homology domain; eNOS, endothelial nitric oxide lyase; ERK, extracellular regulated protein kinase; mTOR, mechanistic target of rapamycin; NF-κB, nuclear factor-κB; NO, nitric oxide; p38 MAPK, mitogen-activated protein kinase; PGC-1α, peroxisome proliferator-activated receptor-γ coactivator-1α; PPAR-α, peroxisome proliferator-activated receptor-α; SIRT1, silent information regulator 1; TNF-α, tumor necrosis factor-α.

APPL1 stimulates insulin signaling through the Akt/mTOR pathway, thereby improving glucose uptake. Akt/mTOR pathway inhibition can prevent apoptosis and minimize myocardial and vascular smooth muscle cell injury [26,33]. APPL1 also activates the p38 MAPK pathway induced by APN and is implicated in glucose uptake [26,34]. The APN signaling pathways and their associated roles are summarized in Figure 2.

## 3. Role of APN in Glucose and Lipid Metabolism Disorders

### 3.1. Regulation of Glucose Metabolism

APN regulates glucose metabolism by protecting β-cells, increasing tissue uptake of glucose, and reducing gluconeogenesis [35].

#### 3.1.1. Protecting β-Cells

Rakatzi et al. discovered that gAcrp at 10 nmol/L partially rescues the β-cell line INS-1 from cytokine- and fatty acid-induced apoptosis, and completely restores autoimmune and lipotoxicity-induced insulin-producing cell dysfunction [36]. Subsequently, Ye et al. demonstrated that APN acts directly on β-cells to increase β-cell proliferation in PANIC-ATTAC mice [37]. The authors of these studies demonstrated that APN regulates metabolism by protecting β-cells, thereby enhancing their viability and regeneration and reducing their apoptosis directly or indirectly.

#### 3.1.2. Increasing Glucose Tissue Uptake

Ceddia et al. were the first to demonstrate that gAcrp increases glucose uptake in skeletal muscle cells via GLUT4 translocation, and reduces the glycogen synthesis rate [38]. Meanwhile, Palanivel et al. were the first to demonstrate that gAcrp and full-length APN mediate cardiomyocyte effects on glucose and fatty acid uptake, and oxidation through AdipoR1 and AdipoR2. Furthermore, APN stimulates glucose uptake and metabolism in cardiomyocytes through actin cytoskeleton remodeling, and the APPL1-dependent AMPK and p38MAPK signaling pathways [39,40]. Furthermore, various studies have shown that APN can increase glucose tissue uptake in skeletal muscles and myocardium.

#### 3.1.3. Reducing Gluconeogenesis

According to Combs et al., a modest rise in circulating Acrp30 (APN) levels can inhibit hepatic gluconeogenic enzyme expression, and endogenous glucose production rate [41]. Ding et al. discovered a novel mechanism by which APN inhibits hepatic gluconeogenesis, and that the APPL1-SirT1-STAT3 pathway mediates APN signaling in primary hepatocytes [42]. The ability of APN to reduce gluconeogenesis remains unexplored, and its mechanism requires further studies.

### 3.2. Regulation of Lipid Metabolism

Nguyen summarized that APN could promote adipocyte differentiation, promote fatty acid (FFA) oxidation and turnover, and regulate lipid metabolism by IR [43].

#### 3.2.1. Promoting Adipocyte Differentiation

Fu et al. found that APN-overexpressing cells differentiate more rapidly into adipocytes, while C/EBP2, PPARγ, and ADD1/SREBP1c expression is enhanced during lipogenesis [44]. Subsequently, Avides et al. expressed and purified human APN in two systems, *Escherichia coli* and baculovirus, which can induce human preadipocyte differentiation; baculovirus produces APN with stronger activity [45]. Yang et al. showed that the effect of APN in promoting preadipocyte differentiation through anti-inflammatory and anti-oxidative stress under inflammatory conditions may be regulated by the PPARγ/Nnat/NF-κB signaling pathway [46].

#### 3.2.2. Promoting Free Fatty Acid (FFA) Oxidation and Clearance

Yoon et al. indicated that APN increases FFA oxidation in skeletal muscle cells by sequentially activating AMPK, MAPK, and PPAR-α [47], while Shetty et al. demonstrated that APN-overexpressing mice show reduced FFA levels, and that APN can stimulate the clearance of FFAs that are likely to enter the oxidative pathway [48]. Lopez-Yus et al. found that APN overexpression in C2C12 cardiomyocytes increased lipid oxidation, and myofiber transition [49].

#### 3.2.3. Insulin Sensitization

**Figure 3:**
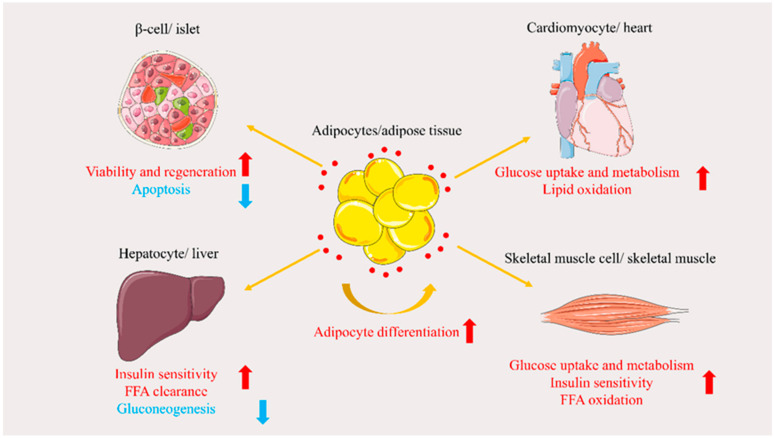
Schematic representation of the role of adiponectin (APN) in glucose and lipid metabolism. Red dots indicate APN, red arrows indicate facilitation, and blue arrows indicate inhibition. Abbreviations: FFA, free fatty acid.

Reduced insulin sensitivity leads to IR [50]. In a cross-sectional study, Moon et al. reported that low APN levels may affect IR [51]. As an insulin-sensitizing adipokine, APN improves IR mainly in the liver and skeletal muscle [52]. Recently, Li et al. revealed two mechanisms by which APN increases insulin sensitivity. First, APN treatment increases white adipose tissue lipoprotein lipase activity, thereby increasing TG absorption into white adipose tissue, and reducing TG storage in the liver and skeletal muscle. Second, APN treatment promotes fatty acid oxidation in skeletal muscle. The two effects of APN can reduce ectopic lipid storage in the liver and muscle, thereby reversing lipid-induced IR [53]. Studies have also explored the insulin-sensitizing effect of APN on adipocytes; for instance, Chang et al. demonstrated that adiponectin deletion impairs insulin signaling, concurrently with reduced AMPK activation in insulin-sensitive 3T3-L1 adipocytes [54]. These findings indicate that APN plays an insulin-sensitizing role in the liver and skeletal muscle; however, its effect on insulin signaling in adipocytes remains to be investigated. APN involvement in the regulation of glucose metabolism and insulin sensitization is illustrated in Figure 3.

## 4. Role of APN in Cardiovascular Disease and Related Advances

The heart has a high energy demand and must, therefore, produce a large amount of adenosine triphosphate (ATP). The heart maintains its energy supply by metabolizing various fuels, including fatty acids, glucose, lactate, ketone, pyruvate, and amino acids, via mitochondrial oxidative phosphorylation [55,56]. The oxidation of (long-chain) fatty acids produces approximately 50–70% of the ATP required by the myocardium. Glycolysis yields <10% of the total ATP production in healthy hearts. Although the heart preferentially uses fatty acids for energy production, it can alter substrates to generate ATP depending on its status to meet the energy requirements [57,58]. In a state of myocardial injury, cardiac energy substrates switch from fatty acids to glucose. However, in an IR state, substrate conversion is no longer possible; hence, transition cannot occur, making fatty acids the only fuel source. This increases lipid uptake and accumulation in the heart, thereby inducing lipotoxicity [59,60,61]. Thus, a balance between lipid degradation and glucose oxidation can improve the CVD associated with glucose and lipid metabolism dysregulation [60].

Glucose and lipid metabolism disorders also lead to inflammation and oxidative stress, which affect cardiovascular homeostasis and cause myocardial damage [14,62]. In glucose and lipid metabolism disorders, lipid accumulation can lead to low-grade chronic inflammation; APN treatment can mitigate inflammation by inhibiting NF-κB-related pathways, thereby downregulating inflammatory factors such as TNF-α and interleukin (IL)-6. The anti-inflammatory effects exerted by APN on macrophages and endothelial cells influence cardiovascular homeostasis [14]. Macrophages can alter their phenotype in response to different stimuli into two polarization states: M1 macrophage polarization is associated with inflammation and tissue destruction, while M2 macrophages have an anti-inflammatory phenotype associated with wound repair and angiogenesis. APN promotes the cellular differentiation of monocytes into M2 macrophages and inhibits their differentiation into M1 [63]. On endothelial cells, APN induces AMPK to regulate vascular homeostasis, and the enhancement of its downstream eNOS activity and subsequent NO production improves endothelial cell function, and blocks the secretion of inflammatory factors [64].

When glucose and lipid metabolism are disturbed, the redox balance of cells is distorted, causing oxidative stress. Reactive oxygen species (ROS) are produced in excess during oxidative stress, and can degrade polyunsaturated fatty acids to generate malondialdehyde. Malondialdehyde can induce toxic stress and cellular DNA damage through mutagenesis [65,66,67]. ROS cause cardiomyocyte death mainly through apoptotic pathways, autophagic pathways, inflammatory pathways, and cytotoxic effects [68]. APN can reduce lipotoxic damage and ROS production through AMPK-related pathways [24,25,26,27].

APN, as a beneficial adipokine for CVD, can affect the balance of glucose and lipid metabolism and inhibit inflammation and oxidative stress, thereby maintaining cardiovascular homeostasis, and reducing myocardial damage.

### 4.1. Atherosclerosis

Atherosclerosis refers to fat and fibrous material accumulation in the innermost arterial intima [69]. Abnormal blood lipid, hyperglycemia, oxidative stress, inflammation, and other abnormal glucose and lipid metabolism are pathological mechanisms underlying atherosclerosis [3]. APN has anti-atherosclerotic effects [7]. Marso et al. found that non-diabetic patients with low circulating APN showed intimal thickening, and increased plaque and plasma lipoprotein levels [70]. Similarly, Csongrádi et al. analyzed intima-media thickness (IMT), considered a marker of initial asymptomatic atherosclerosis, inversely correlated with APN levels in obese subjects [71]. APN can affect atherosclerosis development and complications by regulating lipid metabolism, thereby improving endothelial dysfunction, regulating NO production, and reducing oxidative stress.

#### 4.1.1. Regulation of Lipid Metabolism

Lipid metabolism disorder is the pathological basis for atherosclerosis. APN is downregulated in CVD and associated with various lipoprotein metabolism parameters, including high-density lipoprotein (HDL), TG, and cholesterol. According to Hafiane et al., APN can promote ABCA1-dependent cholesterol efflux to some extent and regulate HDL biogenesis by activating the PPARγ/LXR-α signaling pathway in macrophages [72]. Kobayashi et al. examined the role of HDL in promoting APN gene expression through the CAMKK/CAMKIV pathway via SR-BI/CLA-1 [73]. Studies have also explored the effect of APN on TG metabolism. Qiao et al. observed that APN treatment reduced plasma TG levels by increasing the expression of lipoprotein lipase and very low-density lipoprotein (VLDL) receptor in the skeletal muscle, and reducing VLDL-TG catabolism [74]. However, according to Liang et al., APN upregulates ABCA1 expression in RAW 264.7 macrophages through the LXR-α pathway, promotes cholesterol efflux, and reduces cholesterol content [75]. The above-mentioned studies show that APN can increase serum HD levels, reduce TG levels by enhancing TG-rich lipoprotein catabolism [76], and promote cholesterol efflux, thereby increasing HDL levels. In addition, reduced TG and cholesterol levels can limit atherosclerosis development.

#### 4.1.2. Improvement of Endothelial Dysfunction

Inflammation can directly affect the vascular wall and change endothelial function. Inflammatory reactions are closely associated with atherosclerotic vascular disease progression. Wang et al. revealed that APN could ameliorate NF-κB-mediated inflammatory response, and reduce atherosclerosis progression in apolipoprotein E-deficient mice [77]. Endothelial dysfunction can lead to abnormalities in the fibrinolytic system, which plays an important role in atherosclerotic plaque formation. Plasminogen activator inhibitor-1 (PAI-1) is the main inhibitor of fibrinolysis. Chen et al. provided evidence that APN could inhibit NF-κB binding to the PAI-1 promoter in human umbilical vein endothelial cells through the cAMP/PKA/AMPK signaling pathway, thereby reducing TNF-α-induced PAI-1 expression, and atherosclerotic lesions [78]. Similarly, Mahadev et al. demonstrated that APN plays an important regulatory role in atherosclerosis-related vascular processes by inhibiting VEGF-stimulated HCAEC migration through cAMP/PKA-dependent signaling [79]. Macrophages can promote inflammation and plaque formation, while dead macrophages and cellular debris accumulate to form the core of atherosclerotic necrosis. Ohashi et al. found that APN is a macrophage polarization regulator that facilitates macrophage differentiation to relevant phenotypes, in turn preventing the progression of CVD as observed in cultured mouse and human macrophages [80]. In addition, Tsai et al. demonstrated that rosiglitazone, troglitazone, and Δ2 troglitazone (a novel derivative of troglitazone) could upregulate APN expression and function in human monocytes and macrophages. Monocytes bind to adhesion molecules expressed by activated endothelial cells, which are early pathological manifestations of atherosclerosis. APN expression can inhibit monocyte adhesion to TNF-α-treated endothelial cells by activating the AMPK signaling pathway, affecting the early stages of atherosclerosis [81,82]. In summary, APN improves endothelial dysfunction via the AMPK/NF-κB/TNF-α axis and other signaling pathways, ultimately influencing atherosclerosis development.

#### 4.1.3. Regulation of Nitrous Oxide Production and Oxidative Stress Reduction

Vascular ROS and NO have contrasting activities in atherogenesis. In addition, NO protects against ROS-induced macromolecular damage; ROS limits NO activity. NO synthase catalyzes NO generation in vivo. eNOS is atheroprotective, while iNOS is proatherogenic [83].

Ouedraogo et al. reported that APN reversed hyperglycemia-associated endothelial ROS generation, and protected vascular endothelium via a cAMP/PKA-linked pathway [84]. APN can activate eNOS and inhibit iNOS [85]. In addition, Chen et al. showed that APN could activate eNOS, increase NO production, and prevent the atherosclerosis caused by reduced NO levels through a PI3K-dependent pathway [85]. Similarly, Wang et al. found that APN may protect the aorta from atherosclerotic damage by reducing oxidative stress. Increased eNOS expression in the aorta is one of the possible molecular mechanisms [86]. Cai et al. proposed that APN can activate AMPK through APN receptors and increase ACC phosphorylation, thereby inhibiting iNOS expression and activity, reducing oxidative/nitrative stress and atherosclerotic plaque area, and stabilizing atherosclerotic plaques [87]. APN can potentially ameliorate atherosclerosis by influencing NO and ROS production, and the underlying molecular mechanism remains to be elucidated.

### 4.2. Hypertension

Hypertension is characterized by increased systemic arterial blood pressure (systolic and/or diastolic), which may be accompanied by functional or organic damage to the heart, brain, kidney, and other organs [88]. Hypertension tends to cluster with other atherosclerotic risk factors, such as dyslipidemia, IR, obesity, and oxidative stress [89]. Metabolic abnormalities and metabolic syndrome are strongly associated with the severity of hypertension, and the risk of target organ damage [90,91].

Jung et al. showed that low serum APN levels were associated with an increased risk of new-onset hypertension in men and postmenopausal women [92]. Studies have shown that in ADIPOQ, T45G (rs 2241766) located in exon 2 and G276T (rs 1501299) located in intron 2 are associated with hypertension risk, and circulating APN changes. Wu et al. found that the G276T heterozygous mutation was associated with elevated circulating APN levels and blood pressure, particularly in hypertensive patients [93]. APN levels are tightly correlated with patient blood pressure and the myocardial changes caused by hypertension. Yan et al. showed that low levels of APN and SNP + 45 polymorphisms in the APN gene might play an important role in myocardial fibrosis in hypertensive patients [8].

APN can reduce blood pressure by protecting endothelial cells and promoting NO production. In a study by Ohashi et al., salt-fed APN-deficient (APN-KO) mice developed hypertension with reduced eNOS mRNA levels in the aorta and kidney, and reduced plasma NO metabolites. Hypoadiponectinemia promotes the development of obesity-related hypertension by directly affecting blood vessels, and APN supplementation can reduce blood pressure to some extent in mice [94]. Moreover, Zhiyue et al. indicated that the Fufang Qima capsule could significantly reduce blood pressure, improve pathological vascular changes, and increase NO concentration and eNOS phosphorylation level in the aorta. The antihypertensive and endothelial protective effects of Fufang Qima capsule may be related to activation of the APN/AMPK pathway by upregulation of aortic perivascular adipose tissue and AdipoR2, AMPKα, and phosphorylated AMPKα expression [95].

In recent years, several studies have also shown that APN can lower blood pressure by mediating sodium excretion. For example, Zhao et al. reported that perirenal adipose PPARγ activated by agonists or high sodium intake inhibited renal sodium-glucose cotransporter 2 (SGLT2) function, mediated by increased adipose APN production. The PPARγ/APN/SGLT2 pathway lowers blood pressure by reducing sodium intake, and maintaining glucose homeostasis [96]. In addition, Zhang et al. demonstrated that kidney-specific GRK4 downregulation in hypertensive patients restores APN-mediated sodium excretion, thereby reducing blood pressure in spontaneously hypertensive patients [97]. The above-mentioned results show that APN reduces blood pressure by protecting the endothelium, which promotes NO production and mediates sodium intake or excretion.

### 4.3. Cardiac Hypertrophy

The main function of the heart is to maintain peripheral organ perfusion. The heart and single myocardial cells usually enlarge when the preload or afterload increases. Physiological hypertrophy maintains cardiac function, and pathological hypertrophy can lead to myocardial ischemia and even HF [98]. Hypertrophic hearts experience changes in glucose and lipid metabolism; the heart changes from mainly using fatty acids, to using glucose for energy production. Maintaining normal energy metabolism can reduce cardiomyocyte hypertrophy during cardiac stress [99]. APN exerts a preliminary cardioprotective anti-hypertrophic effect [9] by mitigating cardiomyocyte hypertrophy, and activating related signaling such as AMPK against cardiomyocyte hypertrophy.

#### 4.3.1. Improvement of Myocardial Hypertrophy

Amin et al. demonstrated that endogenous APN protects cardiomyocytes from hypertrophy through a PPARγ-dependent autocrine mechanism using a thiazolidinedione-treated primary cardiomyocyte culture, and a transgenic mouse model expressing a PPARγ constitutive-active version in the heart [100]. Li et al. found that APN promoted HO-1 induction by activating Nrf2 and Brg1, thereby reducing cardiac oxidative stress, improving cardiomyocyte hypertrophy, and preventing dysfunction in patients with diabetes [101]. However, studies have reported different results on the effects of APN on cardiomyocyte hypertrophy. Cardiomyocyte hypertrophy and increased myocardial volume are associated with the hyperactivation of cardiomyocyte enhancer factor-2 (MEF2) family transcriptional regulators. Dadson et al. showed that APN is required to completely induce cardiomyocyte enhancer factor-2 activation in cardiomyocytes, contributing to the cardiac hypertrophy gene expression program in response to pressure overload (PO) [102]. Notably, the regulatory effect of APN on cardiomyocyte hypertrophy remains controversial, as it both improves and induces cardiomyocyte hypertrophy.

#### 4.3.2. Activation of AMPK and Other Related Signaling Pathways

AMPK-related signaling pathways have a non-negligible role in cardiac hypertrophy. Hu et al. demonstrated that AdipeRon, AdipoR1, and AdipoR2, small molecule agonists, can improve isoprenaline (ISO) or L-thyroxine-induced cardiac hypertrophy, and regulate myocardial mitochondrial energy metabolism through AMPK-related pathways [103]. APN also has a positive effect on cardiac hypertrophy resulting from angiotensin II (AngII). Cao et al. found that gAcrp improved AngII-induced cardiac hypertrophy and fibrosis in rat atrial cells by activating the AMPK signaling pathway; wherein, AMPK pivotally interacts with NF-kB and PI3K to mediate the cardioprotective effects of APN [104]. MiR-133a plays a protective role in cardiac hypertrophy. Li et al. showed, for the first time, that APN reverses the miR-133a levels downregulated by AngII via AMPK activation, and reduces extracellular regulated protein kinase1/2 (ERK1/2) phosphorylation in cardiomyocytes [105]. In addition, Li et al. observed that pyridinone could regulate cardiac remodeling of AngII by stimulating APN levels, showing that APN plays an important role in inhibiting pyridinone-induced cardiac hypertrophy, and cardiac fibrosis [106]. In addition to AMPK-related signaling pathways, APN can improve cardiac hypertrophy via other pathways. Leffler et al. studied AdipoRon and demonstrated that it could improve hypertrophy in T2DM sham-operated female rats. Further studies have identified the cardiac APN-Cx43 signaling pathway as a novel target for developing treatments for aggravated cardiomyopathy in women with T2DM [107]. Fujishima et al. demonstrated that APN attenuates AngII-induced cardiac hypertrophic signals, partly through the Akt/GSK3β/β-catenin and Akt/mTOR pathways [108]. The effect of APN on cardiac hypertrophy is mediated via multiple pathways; hence, AMPK requires further investigation.

### 4.4. Myocardial Ischemia/Reperfusion and Infarction

MI/R injury occurs when the blood supply to the heart is interrupted (ischemia) and then re-established (reperfusion) [109]. APN improves MI/R injury by affecting oxidative stress-related signaling pathways.

Tao et al. have demonstrated that APN protects the heart from I/R injury by inhibiting iNOS and nicotinamide adenine dinucleotide phosphate oxidase protein expression, and the resulting oxidative/nitrative stress [10]. Furthermore, Wang et al. provided evidence that the cardioprotective effect of ischemic preconditioning is partly due to APN up-regulation [110]. In contrast, Zhu et al. showed that gAcrp inhibits hypoxia/reoxygenation-induced cardiomyocyte necroptosis and apoptosis by alleviating oxidative stress and p38 MAPK/NF-κB signaling pathway [111]. Activating AMPK is a mechanism by which APN phosphorylates its target proteins. Potenza et al. demonstrated, for the first time, that the protective effects of APN pretreatment on rat hearts are achieved through a signaling pathway involving the AMPK/LKB1/SIRT-1 axis [112]. Zhu et al. demonstrated that APN enhances antioxidant capacity by activating AMPK-dependent STAT3 at Tyr705, and reduces ROS generation by activating AMPK-independent STAT3 at Ser727, conferring cardioprotection. Combining APN and ischemic post-conditioning (IPO) has synergistic protective effects against MI-R injury [113].

Previous studies have shown that APN also has superior protective effects against MI-R in the diabetic state. For example, Wang et al. suggested that hypoadiponectinemia impairs autophagic flux and enhances MI-R injury in the diabetic state. Activating AdipoR restores AMPK-mediated autophagosome formation and antioxidant-mediated autophagosome clearance, and is an effective intervention for MI-R injury in diabetic conditions [114]. Cao et al. showed that the protective effects of I/R injury and IPO are closely related to APN expression in diabetic rats. IPO ineffectiveness is partly due to decreased APN and PI3K/Akt signaling pathway inactivation in diabetic patients [115]. In addition to directly ameliorating MI-R injury via APN, other active components can also indirectly attenuate MI-R injury via the APN pathway. Huynh et al. provided evidence that the CD36-dependent APN pathway mediates the cardioprotective effects of the azapeptide prototype CP-3 (iv) against MI/R. Locally produced APN plays a role in mediating recovery of myocardial function induced by CP-3 (iv) after transient low-flow ischemia [116]. Yang et al. demonstrated that chronic administration of resveratrol up-regulated APN levels and multimerization in T2DM mice, partially alleviating MI/R injury through the APN-AMPK signaling pathway [117]. APN protects the heart from IR injury by reducing oxidative stress and has a better effect in the diabetic state.

Persistent severe myocardial ischemia (MI) leads to myocardial cell death, resulting in myocardial infarction [118]. In a study of individuals aged <60 years, Persson et al. found that low plasma APN concentrations were associated with myocardial infarction. The results remained significant after adjusting for hypertension, high-density lipoprotein cholesterol, smoking, and BMI history [11]. Based on previous studies, Shibata et al. used APN-KO and wild-type mice to cause myocardial infarction by permanent ligation of the left anterior descending artery. They found that APN treatment prevented the development of systolic dysfunction after myocardial infarction by inhibiting myocardial hypertrophy and interstitial fibrosis, and protecting cardiomyocyte and capillary loss [119].

APN also plays a positive role in myocardial infarction in T2DM. Han et al. described that APN could reduce coronary no-reflow injury in T2DM rats by protecting the endothelium, and improving microcirculation and reducing myocardial infarction [120]. Zhang et al. demonstrated in clinical studies that exogenous APN further reduces the no-reflow phenomenon during percutaneous coronary intervention in patients with T2DM and acute myocardial infarction. Exogenous APN can alleviate myocardial and endothelial cell injury, and inhibit inflammation and apoptosis [121]. In summary, APN can prevent myocardial infarction by protecting cardiomyocytes, and improving circulation of myocardial infarction.

### 4.5. Heart Failure

HF, the inability of the heart to provide blood and oxygen required by the surrounding tissues to meet metabolic needs, leads to a clinical syndrome characterized by symptoms such as dyspnea or fatigue, and is the end stage of many CVDs [12,122]. One factor associated with poor prognosis in HF is high APN level, and it has been postulated that increased APN is a compensatory mechanism in HF progression [12]. As a result, APN may serve as a diagnostic and prognostic biomarker in HF.

**Figure 4:**
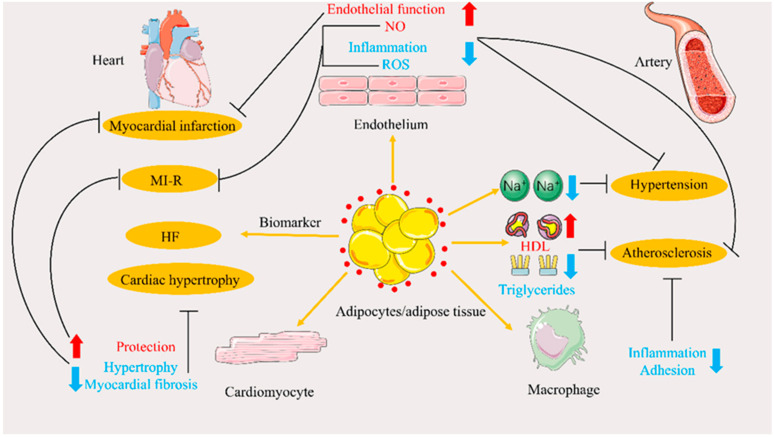
Schematic representation of the role of adiponectin (APN) in cardiovascular diseases related to glucose and lipid metabolism disorders. Red dots indicate APN, red arrows indicate promotion or increase, blue arrows indicate inhibition or decrease, and flat black lines indicate inhibition. Abbreviations: HDL, high-density lipoprotein; HF, heart failure; MI-R, myocardial ischemia/reperfusion; NO, nitric oxide; ROS, reactive oxygen species.

A meta-analysis by Bai et al. suggested that elevated circulating APN levels might be associated with increased all-cause mortality and composite endpoints of death/readmission in patients with acute or chronic HF [123]. Dai et al. demonstrated in clinical trials that APN is a valuable biomarker for acute HF, especially in patients with impaired renal function [124]. In addition to its application in HF clinical diagnosis, APN can be used for HF prognosis. Monzo et al. found that HF patients were more likely to have systemic congestion than other patients; those with congestion-prone status have more severe symptoms and shorter survival. APN is a novel independent congestion-prone status predictor, and can be used as a biomarker in patients with HF [125]. The role of APN in CVD, related to glucose and lipid metabolism disorders, is summarized in Figure 4.

## 5. Problems and Prospects

APN can regulate systemic metabolism and thus affect myocardial metabolism. APN regulates glucose metabolism by protecting β-cells, increasing glucose tissue uptake, reducing gluconeogenesis, and exerting insulin-sensitizing effects mainly in the liver and skeletal muscle cells. The results of the studies on its role in adipocytes are inconclusive. How APN regulates metabolism and insulin sensitization has not been determined yet, with most evidence gathered from in vitro experiments, and no studies exploring its application in clinical practice.

APN plays different roles in CVD associated with glucose and lipid metabolism dysregulation. Atherosclerosis is a multi-stage and complex process, and APN regulates lipid metabolism and improves endothelial function in the initiation of atherosclerosis.

APN also maintains vascular homeostasis and reduces oxidative stress by regulating NO and ROS in atherosclerosis; however, the specific mechanism is unclear. APN itself has a limited ability to modulate atherosclerosis, but other compounds may influence the atherosclerotic process through the APN pathway, and such studies could be further enriched in the future. Several clinical investigations and experimental studies have demonstrated that APN reduces blood pressure by protecting the endothelium, promoting NO production, and mediating sodium intake and excretion. APN-mediated sodium excretion is a novel finding in recent studies, and this pathway-related signaling pathway is independent of AMPK-related pathways. For example, PPARγ/APN/SGLT2-related pathways, which lower blood pressure by reducing sodium intake and glucose homeostasis, suggest that APN plays a unique role in CVD related to glucose and lipid metabolism, and has great research potential.

However, APN does not show benefits in all CVDs, and its effect on cardiomyocyte hypertrophy remains controversial and under-explored. Why do we observe opposite effects of APN associated with cardiac hypertrophy? Both physiological and pathological cardiac hypertrophy initially develop as an adaptive response to cardiac stress [98]. While pathological cardiac hypertrophy has multiple triggers, the animal models of cardiac hypertrophy summarized in this paper are mostly obtained by infusing AngII into SD rats or WT mice, and WT mice are then compared with Ad-KO mice to deeply study the role of APN in cardiac hypertrophy [104,105,106,108]. In addition, cardiac hypertrophy induced by ISO or L-thyroxine injection in SD rats was also observed [103]. In addition to hypertrophic stimuli (AngII, ISO, L-thyroxine, etc.), studies have used SD rats or Wistar rats injected with STZ to induce diabetes, resulting in an indirect model of cardiac hypertrophy caused by diabetes [101,107]. The animal models used in the above studies were more conventional and the triggers were also relatively clear, mainly focusing on hypertrophic stimuli and diabetes. There are two specific transgenic mouse models, a transgenic mouse model that expresses a constitutive-active version of PPARγ (CA-PPARγ) in the heart [100], and well compound sensor characterized transgenic MEF2 “mice” (MEF2-LacZ) with Ad-KO to create MEF2LacZ/Ad-KO mice [102]. The former (CA-PPARγ) was protected from high-fat diet-induced cardiac hypertrophy [100]. A high-fat diet, similar to diabetes, indirectly induces cardiac hypertrophy. Whereas cardiac hypertrophy induced by PO in the latter, which is induced by transverse aorta constriction (TAC), is associated with excessive activation of transcriptional regulators of the MEF2 family. The article demonstrated that APN signaling is required for cardiac MEF2 activation by PO [102]. In the relationship between APN and cardiac hypertrophy summarized in this paper, APN only plays a promoting role in cardiac hypertrophy caused by PO. However, by reviewing other literatures, we found that the role of APN in pressure overload-induced cardiac hypertrophy is also ambiguous. O’Shea’s team showed that APN deficiency had no effect on left ventricular hypertrophy in TAC mice [126]. Similarly, Hecker et al. found that Ad-KO mice affected by abdominal aortic binding (a way to cause cardiac PO) and a high-fat diet did not accelerate cardiac hypertrophy [127]. However, Shimano et al. demonstrated that Ad-KO mice exhibited greater cardiac hypertrophy following TAC surgery, compared to WT mice [128]. Han’s team also showed that treatment with APN reduced cardiac hypertrophy in TAC mice [129]. This is an interesting phenomenon, suggesting that APN and its derivatives should focus on the causes of cardiac hypertrophy if used in the treatment of cardiac hypertrophy in subsequent studies. For example, there is no clear conclusion on the mechanism by which APN affects the development of cardiac hypertrophy induced by pressure overload. Because the type of cardiac hypertrophy stimulation and the nature of downstream signaling mechanisms largely determine the fate of cardiac hypertrophy [98], further study of the effect of APN on cardiac hypertrophy with different stimulation types may be a breakthrough in the treatment of cardiac hypertrophy with APN.

In addition, APN relies on AMPK-related pathways to exert antioxidant and reduced oxidative stress effects to attenuate MI-R injury. It also reduces further myocardial infarction development. Moreover, APN plays an important role in MI-R injury, and myocardial infarction in the context of T2DM. The specific internal mechanism remains to be elucidated. Finally, several studies have shown that APN application in HF does not affect the treatment; however, it has a better indicative role in HF diagnosis and prognosis, and hence is an HF biomarker. The role played by APN in CVD related to glucose and lipid metabolism disorders is summarized in Table 1.

APN is secreted by adipose tissue and modified to become multimers into the circulation. APN binds to its receptors AdipoR1 and AdipoR2, and initiates a series of signal transduction events that function in target organs or target tissues [15]. If exogenous APN is not supplemented in vitro and only exerts its biological efficacy through endogenous APN, it can exert its effect through the following methods: (1) Increasing APN-related gene expression and APN secretion; the amount of APN entering the circulation is also increased. (2) Activating AdipoR1 and AdipoR2 via agonists to initiate downstream signaling pathways. (3) Enhancing the transduction of signaling pathways mediated by APN. APN is a relevant target for the treatment of CVD associated with glucose and lipid metabolism disorders, and has a diverse pathway compared with traditional targets and provides more options for the development of related drugs. However, similarly, APN as an endogenous secretion has a unique biological macromolecular structure and a complex biological environment in vivo, and it is difficult to thoroughly elucidate its mechanism of action. Recently, some therapeutic methods to increase APN secretion and modification have achieved minor beneficial therapeutic effects owing to their polysomal structure, and high serum concentrations. Overall, APN presents both opportunities and challenges in CVD associated with the dysregulation of glucose and lipid metabolism.

A key signal for the role of APN in CVD is AMPK, which plays a key role in regulating anabolic pathways related to energy expenditure by responding to changes in cellular energy status and ATP production/consumption [130], mediating glucose and lipid metabolism, mitochondrial biogenesis, inflammation, oxidative stress, cell proliferation and apoptosis, and cell hypertrophy. AMPK signaling pathway networks should be explored in future studies. Using AMPK as an entry point, APN plays a role in a variety of metabolic target organs and target tissues, including the skeletal muscle, liver, islets, endothelial vessels, myocardium, and adipose tissue. In the future, attention should be paid not only to the interactive effects of APN between signaling pathways at the molecular level, but also to the effects produced after APN circulates across various tissues and organs, and the interactive effects.

Associated drug development can also be carried out by simulating the physiological functions of APN in various targeted tissues and organs. Since most current research on APN has been conducted using cell and rodent models, which cannot fully reflect human physiology, clinical studies on APN and its derivatives should be carried out based on preclinical experiments in the future.

Abnormal glucose and lipid metabolism is a key CVD trigger, suggesting further exploration of the role of APN in diabetes-induced cardiovascular complications could be beneficial. However, the role of APN in CVD is conflicting, as illustrated in our summary of the effect on cardiac hypertrophy. In fact, the paradoxical role of APN in cardiovascular dysfunction has been explained [131], which may be related to the circulating levels of APN. The circulating levels of APN are influenced by the complex balance between APN production and clearance, in addition to complex physiological and pathological conditions in the human body. To truly design a regimen for the treatment of CVD, related to glucose and lipid metabolism through APN-related pathways, it is necessary to answer the following questions: (1) Is the effect of APN on CVD in the human body entirely positive? Is there a negative effect, and what is the specific mechanism? (2) Is there a compensatory response?

APN has a few biomarker attributes, is associated with disease progression, is easily measured in plasma or serum, and can be quantified using cost-effective, reliable, and reproducible assays. However, the inclusion of APN as a potential biomarker in clinical guidelines and practice is fraught with challenges associated with experimental design, sample quality, data measurement and analysis, and the high costs associated with clinical trials assessing efficacy. Nevertheless, the applicability of APN as a biomarker for CVD diagnosis and prognosis can be further explored in the future to address the design- and analysis-associated challenges in clinical practice, and reduce costs [132].

## 6. Conclusions

APN improves cardiac metabolism by regulating glucose and lipid metabolism, and increasing insulin sensitivity. APN protects the cardiovascular system by protecting myocardial cells, improving endothelial cell function, reducing oxidative stress and inflammation, slowing cardiovascular system diseases with glucose and lipid metabolism disorders as the main triggers, including atherosclerosis, hypertension, myocardial hypertrophy, myocardial ischemia, and myocardial infarction. APN could also be a potential biomarker for the diagnosis and prognosis of conditions such as heart failure. Most studies on APN mechanism are based on in vitro experiments. Therefore, in vivo experiments and clinical studies remain warranted. In addition, how APN systematically acts in the human body remains to be elucidated, and the different or even diametrically opposite effects it shows in the cardiovascular system remain to be explored. Furthermore, its development and application as a biomarker should be closely integrated into clinical practice. The use of APN and its derivatives in clinical settings for CVD management is not yet feasible; hence, further research is required in clinical settings.

## Figures and Tables

**Table 1:** Adiponectin (APN) in cardiovascular diseases related to glucose and lipid metabolism disorders.

Disease	Effect of APN	References
Atherosclerosis	Induces increased serum HDL levels, reduces serum triglyceride levels, and promotes cholesterol efflux.	[72,73,74,75,76]
	Improves endothelial dysfunction and plays an anti-inflammatory role via the AMPK/NF-κB/TNF-α and other signaling pathways.	[77,78,79,80,81,82]
	Activates eNOS and inhibits iNOS to regulate NO production and maintain cardiovascular homeostasis. Reduces ROS production to reduce oxidative stress	[84,85,86,87]
Hypertension	Increases NO concentration, protects vascular endothelium, and mediates sodium intake and excretion to lower the blood pressure	[94,95,96,97]
Cardiac hypertrophy	The effects of improving and inducing cardiomyocyte hypertrophy have both been reported.	[100,101,102]
	Improves cardiac hypertrophy and myocardial fibrosis induced by multiple factors mainly mediated by AMPK.	[103,104,105,106,107,108]
Myocardial Ischemia/reperfusionand infarction	Reduces oxidative stress and protects the heart in MI/R; works in T2DM state.	[10,110,111,112,113,114,115,116,117]
	Protects myocardial cells and improves microcirculation to prevent the occurrence and development of myocardial infarction; it also plays a role in T2DM state.	[11,119,120,121]
Heart failure	Biomarker for diagnosis and prognosis.	[123,124,125]
